# Novel ALK inhibitors in clinical use and development

**DOI:** 10.1186/s13045-015-0122-8

**Published:** 2015-02-27

**Authors:** Chaitanya Iragavarapu, Milaim Mustafa, Akintunde Akinleye, Muhammad Furqan, Varun Mittal, Shundong Cang, Delong Liu

**Affiliations:** Department of Medicine, Westchester Medical Center and New York Medical College, Valhalla, NY 10595 USA; Department of Medicine, Division of Hematology and Oncology, University of Iowa Carver College of Medicine, Iowa City, IA 52242 USA; Department of Medicine, Division of Hematology/Oncology, Albert Einstein Medical Center, Philadelphia, PA 19141 USA; Department of Oncology, Henan Province People’s Hospital|, Zhengzhou University, Zhengzhou, China; Henan Cancer Hospital, Zhengzhou University, Zhengzhou, China

**Keywords:** Anaplastic lymphoma kinase, ALK-1, Crizotinib, Ceritinib

## Abstract

Anaplastic lymphoma kinase 1 (ALK-1) is a member of the insulin receptor tyrosine kinase family. ALK-1 was initially found in anaplastic large cell lymphoma (ALCL). ALK mutations have also been implicated in the pathogenesis of non-small cell lung cancer (NSCLC) and other solid tumors. Multiple small molecule inhibitors with activity against ALK and related oncoproteins are under clinical development. Two of them, crizotinib and ceritinib, have been approved by FDA for treatment of locally advanced and metastatic NSCLC. More agents (alectinib, ASP3026, X396) with improved safety, selectivity, and potency are in the pipeline. Dual inhibitors targeting ALK and EGFRm (AP26113), TRK (TSR011), FAK (CEP-37440), or ROS1 (RXDX-101, PF-06463922) are under active clinical development.

## Introduction

Anaplastic lymphoma kinase 1 (ALK-1) is a member of the insulin receptor tyrosine kinase family (RTK) [1]. Members of this family include α and β type PDGF receptors, EGF receptor, HER2/neu, insulin and IGF-1 receptors which regulate cellular growth and may trigger neoplastic transformation when mutated, translocated, or expressed aberrantly [1-3]. ALK-1 first was found to be associated with the (2; 5)(p23; q35) chromosome translocation in Ki-1 lymphoma or anaplastic large cell lymphoma (ALCL) [4]. The same translocation has also been associated with Hodgkin lymphoma [1]. Multiple mutations involving the ALK gene have since been identified in ALCL. ALK mutations have also been implicated in the pathogenesis of rhabdomyosarcoma [5], inflammatory myofibroblastic pseudo tumor [6], neuroblastoma [7] and non-small cell lung Cancer [8]. In this article, we discussed common ALK mutations and provided a review of ALK-1 Inhibitors that are currently in clinical use or under clinical development.

## ALK-1 mutations and oncogenesis

Multiple mutations involving the ALK gene located on 2p23 have been described. The first and prototype of these mutations has been the NPM-ALK mutation caused by translocation (2; 5)(p23; q35) [4,9,10]. This mutation fuses the nucleophosmin (NPM) gene with the ALK gene and was first described in Ki-1 Lymphoma. Ki-1 Lymphoma is a distinct subset of large cell lymphomas that are characterized by CD-30 (Ki-1 antigen) positivity. CD30 is a transmembrane protein which belongs to the nuclear growth factor superfamily and is thought to be involved in ligand binding [4]. NPM encodes for the nucleophosmin protein that is localized to the nucleolus and involved in ribosomal assembly. It is postulated that it provides positive feedback to cell growth [11,12]. The NPM-ALK fusion gene encodes a chimeric receptor tyrosine kinase (RTK) that is de-regulated and constitutionally activated. This leads to activation of phospholipase C-γ (PLC-γ) [8]. Activation of PLC-γ leads to growth factor independent proliferation of lymphocytes. Another mechanism that has been elucidated is the hyperphosphorlyation of p80. Fusion of ALK with NPM leads to hyperphosphorylation of p80 and its constitutional activation. This constitutionally active p80 is localized to the cytoplasm and catalyzes the phosphorylation of SH2 domain-containing transforming protein (SHC), an adaptor protein, and insulin receptor substrate 1 (IRS-1) with downstream effects on RAS and epidermal growth factor receptor (EGFR) pathways [12].

Other mechanisms that have been unearthed mainly occur through the Jun set of proteins [13,14]. Jun (cJun, JunB and JunD) are members of the activated protein 1 (AP-1) transcription factor complex. cJun is regulated by the NPM-ALK tyrosine kinase via pathologic phosphorylation and subsequent activation of cJun N-terminal kinase (JNK), the protein kinase capable of phosphorylating serine residues in the N-terminal of cJun and effecting its subsequent activation [13]. JNK is only physiologically phosphorylated by the mitogen activated protein kinase (MAPK) kinases MKK4 and MKK7. However, in the ALCL cells, JNK is phosphorylated by NPM-ALK which in turn phosphorylates and activates cJun.

Activated cJun causes the transcriptional activation of cell cycle proteins (Cyclin D1, Cyclin D3, Cyclin A and Cyclin E) and the inhibition of tumor suppressors such as p53, p21^Cip1^ and p16^Ink4^. This is mediated through the recruitment of cAMP response element binding (CREB) protein (CBP) activator [13]. JunB, another member of the Jun subset of AP–1 complex, is also a positive regulator of cell cycle progression [14]. NPM-ALK also increases JunB expression through the mTOR pathway. mTOR is activated by the phosphoinositol 3- kinase/Akt pathways [14,15].

NPM-ALK has also been shown to act through the signal transducer and activator of transcription (STAT), principally STAT3 and STAT5 [16-19]. STAT3, for example, is constitutionally activated by NPM-ALK phosphorylation and is actively involved in the malignant transformation of NPM-ALK expressing lymphocytes [17]. Activated STAT3 enhances the positive autocrine loop involving IL-6 and the IL-6 receptor (IL6R), which in turn up-regulates the expression of Bcl-xL and survivin, two anti-apoptotic factors [18]. STAT5 activation also is thought to protect cells from apoptosis, likely from activation of anti-apoptotic factors such as A1 (or its human homologue, Bfl-1), Bcl-xL, pim-1 and oncostatin M [16].

Another mechanism for NPM-ALK oncogenesis has been elucidated as occurring through the phosphorylation of p60^c-src^. p60^c-src^ is a src kinase which plays specific roles in downstream effects of the T-cell receptor and causes hematopoietic growth factor independence specifically of IL-3 and granulocyte-macrophage colony stimulating factor (GM-CSF) [20]. Activated Src kinase can lead to activation of NPM-ALK with downstream effects on PI3K/Akt. The effect of ALK on PLC-γ, Shc, IRS-1 and PI3K has been shown to be mediated through pleiotrophin, the ligand for the ALK receptor [21].

Apart from the NPM-ALK mutation, TPM3-ALK mutation caused by the (1;2)(q25;p23) translocation fused ALK with TPM3 gene located on 1q25 [22-24]. TPM3 encodes a non-muscular tropomyosin protein. Tropomyosins are actin binding proteins that mediate the effect of ionized calcium on actin-myosin interaction in skeletal muscle cells [22]. TPM3 has been shown to be fused with the NTRK1 tropomyosin receptor kinase in ALCL and papillary thyroid cancers [22,25,26]. Another tropomyosin gene, TPM4, has also been found to be fused to the ALK gene in inflammatory myofibroblastic tumors (IMT) and other tumors [24,27-30].

Another ALK mutation from fusion of ALK to the ATIC gene has been described [31,32]. ATIC gene encodes the 5-aminoimidazole-4-carboxamide ribonucleotide formyltransferase/IMP cyclohydrolase (AICARFT/IMPCH) bifunctional enzyme. This enzyme catalyzes the last two steps in the purine synthesis pathway. The fusion gene becomes constitutionally active, leading to pathologic activation of ALK. Additional mutations identified in both solid tumors and hematological malignancies include MSN-ALK, MYH9-ALK, RANBP2–ALK, CARS-ALK, CLTCL-ALK [3,33-44]. Rare mutations have been described in NSCLC, lymphoma, renal cell carcinoma and colon cancer [37,45-58] (Tables 1 and 2).

**Table 1:** **Chromosomal translocation and fusion proteins in solid tumors involving ALK gene**

**Disease**	**Chromosomal rearrangement**	**Fusion protein**	**Frequency (%)**	**Reference**
NSCLC	inv(2)(p21;p23)	EML4-ALK	2-5	[59,64,65]
	t(2;3)(p23;q21)	TFG-ALK	2	[65]
	t(2;10)(p23;p11)	KIF5B-ALK	<1	[53,57]
	t(2;14)(p23;q32)	KLC1-ALK	<5	[55]
	t(2;9)(p23;q31)	PTPN3-ALK	ND	[50]
IMT	t(1;2)(q25;p23)	TPM3-ALK	0.5	[24]
	t(2;19)(p23;p13)	TPM4-ALK	<5	[24]
	t(2;17)(p23;q23)	CLTC-ALK	<5	[40,42,43]
	inv(2)(p23;q35)	ALK-ATIC	<5	[32]
	t(2;11;2)(p23;p15;q31)	CARS-ALK	<5	[34,35]
	t(2;2)(p23;q13)	RANBP2-ALK	<5	[36]
	inv(2)(p23;p15;q31)	RANBP2-ALK	<5	[38]
	t(2;4)(p23;q21)	SEC31L1-ALK	<5	[52]
BC	inv(2)(p21;p23)	EML4-ALK	<5	[64]
CRC	inv(2)(p21;p23)	EML4-ALK	<5	[64]
	t(2;2)(p23.3)	C2orf44-ALK	<5	[51]
ESCC	t(2;19)(p23;p13)	TPM4-ALK	ND	[27,28]
RCC	t(2;10)(p23;q22)	VCL-ALK	ND	[47]
	t(1;2)(q25;p23)	TPM3–ALK	ND	[23]
	inv(2)(p21;p23)	EML4–ALK	ND	[23]

NSCLC; non small cell lung cancer, IMT; inflammatory myofibroblastic tumor, BC; breast cancer, CRC; colorectal cancer, ESCC; esophageal squamous cell carcinoma, RCC; renal cell carcinoma, ND; not determined.

**Table 2:** **Chromosomal translocations and fusion proteins in hematologic malignancies involving ALK gene**

**Disease**	**Chromosomal rearrangement**	**Fusion protein**	**Frequency (%)**	**Reference**
ALCL	t(2;5)(p23;q35)	NPM-ALK	75-80	[9]
	t(2;17)(p23;q25)	ALO17-ALK	<1	[34]
	t(2;3)(p23;q21)	TFG-ALK	2	[48]
	t(2;X)(p32;q11-q12)	MSN-ALK	<1	[44]
	t(1;2)(q25;p23)	TPM3-ALK	12-18	[22]
	t(2;19)(p23;p13)	TPM4-ALK	<1	[30]
	inv(2)(p23;q35)	ATIC-ALK	2	[31]
	t(2;22)(p23;q11.2)	MYH9-ALK	<1	[37]
	t(2;17)(p23;q23)	CLTCL-ALK	2	[58]
DLBCL	t(2;5)(p23;q35)	NPM-ALK	ND	[10]
	t(2;17)(p23;q23)	CLTC1-ALK	ND	[41]
	t(2;5)(p23.1;q35.3)	SQSTM1-ALK	ND	[54]
	ins(4)(2;4)(p23;q21)	SQSTM1-ALK	ND	[56]
	t(2;4)(p24;q21)	SEC31A-ALK	ND	[45]
HL	t(2;5)(p23;q35)	NPM-ALK	ND	[1]

ALCL; anaplastic large cell lymphoma, DLBCL; diffuse large B cell lymphoma; HL: Hodgkin lymphoma; ND; not determined.

EML4-ALK fusion gene was initially identified in 2007 in non-small cell lung cancer (NSCLC) [59]. This has facilitated the development of the first ALK inhibitor, crizotinib [60]. This mutation arises from inv(2)(p21p23) which leads to the fusion of echinoderm microtubule-associated protein like-4 (EML4) gene with ALK gene. The fusion protein plays a pivotal role in the malignant transformation of susceptible lung parenchyma [61]. EML4 is a member of the EML protein (EMAP) family and plays an important role in the correct formation of microtubules [62]. The EML4-ALK fusion kinase has an ALK fragment identical to the ALK fragment in NPM-ALK. This intracellular kinase is bound to the amino-terminal coiled-coil domain of EML4 and is thought to be responsible for the transforming activity of the fusion protein [23,63-65].

## ALK inhibitors in clinical use

Two small molecule inhibitors, crizotinib and ceritinib, of ALK kinase are in clinical use now, several more are in active clinical development (Table 3).

**Table 3:** **ALK inhibitors in clinical use and development**

**Drug**	**Phase**	**Tumors**	**Common toxicities**	**Reference**
Crizotinib	III	NSCLC	Visual disturbance, nausea, vomiting, constipation, edema	[68-71]
Alectinib	II/III	NSCLC, ALCL, neuroblastoma	Neutropenia, elevated CPK	[79-82]
Ceritinib	II/III	NSCLC	Diarrhea, elevated transaminases	[86-91]
AP-26113	I/II	NSCLC	Nausea, fatigue, diarrhea	[92-94]
ASP-3026	IB	NSCLC	Nausea, vomiting, constipation, abdominal pain	[95,96]
X-376	Preclinical	NSCLC, ALCL, neuroblastoma cell lines	N/A	[98]
X-396	Preclinical	NSCLC, ALCL, neuroblastoma cell lines	N/A	[98]
TSR-011	I/II	NSCLC, pancreatic, ovarian, and salivary gland cancers	Dysaesthesia, QTc prolongation	[99]
CEP-37440	I	NSCLC, SCCHN, colorectal, pancreatic, prostate, and breast cancers	Nausea, vomiting, diarrhea headaches	[100]
NMS-E628	I/II	advanced solid tumors	N/A	[102]
PF-06463922	I/IIA	NSCLC	N/A	[103]

ALCL-anaplastic large cell lymphoma; CPK-creatine phosphokinase; N/A-not available; NSCLC-non-small cell lung cancer; SCCHN-squamous cell cancer of head and neck.

### Crizotinib

Crizotinib (PF-02341066, Xalkori, Pfizer) is an orally active small molecule inhibitor of ALK, c-MET/hepatocyte growth factor receptor (HGFR) kinase and ROS1 receptor tyrosine kinase [66,67]. Since August 2011, crizotinib has been approved for treatment of locally advanced or metastatic NSCLC that are ALK positive [60,68-70].

The maximum dose reached in the phase I dose escalation trials for crizotinib was 250 mg twice daily, which was therefore selected for an expanded cohort of 82 patients with advanced ALK-positive NSCLC [70]. Out of 82 patients, 46 had confirmed partial response (PR) and 1 confirmed complete response (CR) with an impressive overall response (OR) of 57%. The estimated progression free survival (PFS) was 72%. These results were upheld in an updated report in which 87 of 143 patients had an OR of 60.8% (95% CI 52.3-68.9), including 3 CR and 84 PR. Median PFS was 9.7 months (95% CI 7.7-12.8), estimated overall survival at 6 and 12 months was 87.9% (95% CI 81.3-92.3) and 74.8% (66.4-81.5) respectively. Most common drug related adverse events were grade 1 or 2, including visual effects, nausea, vomiting, constipation, diarrhea and peripheral edema. The most common grade 3 and grade 4 adverse events were neutropenia (n = 9), elevated alanine aminotransferase (n = 6), hypophosphatemia (n = 6), and lymphopenia (n = 6) [68]. PROFILE 1005 was a global, multicenter, open label, single arm phase 2 study evaluating safety and efficacy of crizotinib (250 mg oral bid every 3 weeks) in patients with advanced ALK positive NSCLC who progressed after more than one cycle of chemotherapy [69]. Of the 255 patients evaluated for tumor response, ORR was 53% (95% CI: 46–60) and disease control rate at 12 weeks was 85% (95% CI: 80–89). Median PFS was 8.5 months (95% CI: 6.2-9.9) and median duration of response was 43 weeks (96% CI: 36–50). PROFILE 1007, a phase 3 study comparing crizotinib to standard chemotherapy (premetrexed or docetaxel) was updated in 347 patients previously treated with first line platinum based chemotherapy. The median PFS was 7.7 months in crizotinib group (n = 173) compared to 3.0 in chemotherapy group (n = 174), hazard ratio of crizotinib to chemotherapy was 0.49 (95% CI: 0.37-0.67); p < 0.001). The ORR was 65% (95% CI: 58–72) with crizotinib compared to 20% (95% CI: 14–26) with chemotherapy (p < 0.001) [60]. Currently another phase 3 study, PROFILE 1014, is evaluating crizotinib vs. chemotherapy in patients with advanced ALK positive NSCLC patients as a first line therapy [71]. At the last update in ASCO 2014 annual meeting, 343 pts with untreated advanced non-squamous *ALK*-positive NSCLC were treated with either crizotinib 250 mg PO BID (n = 172) or PPC (pemetrexed 500 mg/m^2^ + either cisplatin 75 mg/m^2^ or carboplatin AUC 5–6; all IV q3w for <=6 cycles; n = 171). The primary endpoint was PFS. After disease progression, crossover to crizotinib was allowed for pts on PPC. Superiority of crizotinib over PPC in prolonging PFS (median 10.9 vs. 7.0 mo; HR: 0.454; 95% CI: 0.346–0.596; P < 0.0001) was reported. Crizotinib showed higher ORR than PPC (74% vs. 45%; P < 0.0001) [71].

Unfortunately majority of patients invariably develop resistance to crizotinib during the first year [60,68]. Mechanism of acquired resistance to crizotinib can be classified into three well-recognized categories. These are the development of new ALK domain mutations; amplification of the EML4-ALK gene and activation of alternating pathways that bypass the ALK pathway. Two secondary mutations, L1196M and C1156Y, within the kinase domain of EML4-ALK in tumor cells were reported in a patient during the relapse phase of treatment with an ALK inhibitor. These developed independently in subclones of the tumor cells and conferred marked resistance to two different ALK inhibitors [46]. L1196M gatekeeper mutation results from substitution of leucine by methionine at position 1196 of the ALK kinase domain. This mutation alters the ATP-binding site of ALK and interferes with the binding of inhibitor. C1156Y mutation in the ALK domain involves substitution of a cysteine by tyrosine at position 1156. Several other mutations of ALK kinase domain have been reported so far, G1269A, L1152R, G1202R, 1151Tins and S1206Y [72-76]. The second well established mechanism of crizotinib resistance is the amplification of EML4-ALK fusion gene through two methods, more copies per cell and more cells displaying the rearrangement pattern [72,73]. The third category of acquired resistance to ALK agents represents the activation of alternating signaling pathways bypassing ALK. The EML4-ALK fusion protein is one of the client proteins for heat shock protein 90 (Hsp90), which is a molecular chaperone that regulates the correct folding, stability, and function of numerous client proteins. Ganetepib, which is an Hsp90 inhibitor, has been shown to be effective in patients with secondary ALK mutations and in tumor cells with ALK amplification [77]. Hsp90 inhibition has been shown to cause regression of EML4-ALK driven xenograft of lung adenocarcinomas [78]. EGFR up-regulation has also been observed in ALK positive tumor cell lines resistant to crizotinib [72,74]. These mechanisms can occur independently, or simultaneously, suggesting that the combination of both ALK and EGFR inhibitors may represent an effective therapy for this specific subset of NSCLC patients [72,74]. KIT gene amplification has also been identified as a potential bypass signaling pathway in crizotinib resistant patients. This resistance mechanism likely involves support by the cancer stroma since stem cell factor (SCF), the KIT ligand, is produced specifically in the stroma of resistant cancer cells with KIT amplification [72].

Therefore, there is a unmet need for novel ALK inhibitors that can overcome these resistance mechanisms. The development of a range of new ALK inhibitors is underway both in pre-clinical and clinical studies.

#### Alectinib (CH-5424802, AF-802, RO05424802)

Alectinib, also known as AF-802, CH-5424802 or RO-5424802, is a highly selective, orally bioavailable ALK inhibitor with ten-fold greater potency than crizotinib in kinase assays (IC_50_, 1.9nM) [79,80]. A carbonitrile derivative, alectinib has potent efficacy against ALK addicted tumors, such as NSCLC expressing EML4-ALK, ALCL expressing NPM-ALK, and ALK amplified neuroblastoma [79]. The compound has also exhibited substantial inhibitory property against mutant ALK enzymes including ALK L1196M, ALK F1174L, and ALK R1275Q [79].

In July 2014, alectinib was granted approval in Japan for the treatment of patients with recurrent/relapsed ALK+ NSCLC [81]. Similarly, the compound has gained Breakthrough Therapy Designation (BTD) by the U.S. FDA in patients with ALK+ NSCLC who had progressed on crizotinib. In an initial phase I dose-escalation portion of the AF-001JP study, treatment of 24 crizotinib-naïve patients with recurrent/relapsed ALK+ NSCLC with alectinib at doses ranging from 20–300 mg twice daily was found to be safe and well tolerated [81]. No DLTs were observed and 300 mg twice daily was chosen as the recommended phase II dose [81]. In the subsequent expanded phase II part of the trial, the agent exhibited clinical activity achieving an ORR of approximately ninety-four percent including 2 CR and 41 PR in 46 patients evaluable for response [81]. The most serious adverse events noted were neutropenia and elevated creatine phosphokinase levels [81]. Similarly, alectinib has demonstrated promising antitumour activity in patients with ALK-rearranged NSCLC resistant to crizotinib, including those with CNS metastases. The phase I/II study conducted by Gadgeel and colleagues showed that alectinib was associated with acceptable toxicity profile in patients who progressed on or were intolerant to crizotinib [82]. No new safety signal emerged; however grade 3 headaches and neutropenia were identified as DLTs in a cohort of patients who received alectinib 900 mg twice a day [82]. The ORR in 44 out of forty-seven patients evaluable for response was 55%, including 1 CR and 23 PR, and sixteen patients had stable disease after a median follow-up of 126 days [82] . Interestingly, a subset analysis of 21 patients with CNS metastases at baseline enrolled in the study showed a disease control rate of approximately 90%. In contrast to the precedent study [81], this recent trial chose alectinib 600 mg twice a day as the recommended dose for subsequent phase 2 studies [82]. Given these favorable results, ALEX, a phase III randomized trial has been initiated to compare alectinib with crizotinib in treatment-naïve ALK-positive advanced NSCLC patients. The study primary endpoint is PFS and the estimated completion date is December 2017 [NCT02075840]. Alectinib was recently reported to be active in crizotinib-resistant NSCLC patients with leptomeningeal metastasis [83].

#### Ceritinib (LDK378)

Ceritinib (LDK378, Celgene) is a potent and selective small molecule tyrosine kinase inhibitor of ALK [84,85]. Ceritinib was shown to be active in NSCLC resistant to crizotinib [86-88]. Preliminary results of a multicenter phase I study of LDK378 were presented at the 48th ASCO annual meeting [89]. Among the 131 patients with advanced malignancies harboring a genetic alteration in ALK, 59 patients were enrolled in the dose escalation phase during which a maximum treatment dose (MTD) of 750 mg was established, and 72 patients in a dose expansion cohort at MTD. In the 123 NSCLC patients, median PFS was 8.6 months (95% CI, 4.3-19.3). In 88 evaluable NSCLC patients who received LDK378 at doses of 400–750 mg daily, the overall response rate (ORR) was 70%. ORR was 73% in a subset of 64 patients who had developed crizotinib resistance. LDK378 was well tolerated. There was no treatment related death and the most common grade 3/4 adverse effects were elevated ALT (12%), diarrhea (7%) and AST elevation (6%). These were updated in a recent publication [90]. Currently two phase II studies are undergoing, the first (NCT01685060) evaluating LDK378 in ALK activated NSCLC patients previously treated with chemotherapy and crizotinib; and the second (NCT01685138) in ALK activated NSCLC patient’s naïve to crizotinib +/–chemotherapy. A Phase III multicenter, randomized study (NCT01828099) is evaluating LDK378 versus standard chemotherapy in previously untreated adult patients with ALK-positive, stage IIIB or IV, NSCLC. Another phase III study is evaluating the antitumor activity of ceritinib versus chemotherapy in patients previously treated with platinum based chemotherapy and crizotinib (NCT01828112). Ceritinib has recently been approved for use in NSCLC patients who have progressed on or are intolerant to crizotinib [91].

### Novel ALK inhibitors in clinical development

Multiple new ALK inhibitors are being developed, each with its own unique set of characteristics as detailed below.

#### AP26113

AP26113 is a potent and selective ALK inhibitor [92]. AP26113 induced tumor regression in BaF3 xenograft model expressing EML4-ALK, and EML4-ALK harboring G1269S and L1196M (gatekeeper) mutations. In preclinical studies, AP26113 was shown to be active against all 9 clinically-identified crizotinib-resistant mutants tested [93]. AP26113 is also a potent, reversible inhibitor of activated and T790M-mutant EGFR, yet it does not inhibit the native enzyme [94]. A phase I/II study was initiated (NCT01449461) to evaluate AP26113 as a dual ALK/mutant EGFR inhibitor. As of 14 Jan 2013, 44 patients were enrolled including 37 with NSCLC [94]. In the dose escalation phase (30-300 mg), two dose limiting toxicities were observed, grade 3 ALT elevations at 240 mg and grade 4 dyspnea at 300 mg. The recommended phase II dose was identified as 180 mg. The most common adverse effects were nausea (45%), fatigue (39%), diarrhea (27%). Among 18 evaluable ALK+ patients, four out of 5 patients with CNS lesions showed improvement on follow up imaging, including one patient resistant to crizotinib and ceritinib. Sixteen patients had EGFR mutation (EGFRm). Of 12 patients with EGFRm, one patient responded at 120 mg (duration 21 weeks, ongoing) and 6 patients had stable disease (2 ongoing, duration 7 and 31 weeks, respectively). In a later update, 114 pts were enrolled: 65 in phase 1 (30–300 mg) and 49 in phase 2 (180 mg) [93]. There were 106 pts with NSCLC. The most common treatment-emergent AEs (20%) were similar to the previous report. Early onset of pulmonary symptoms (dyspnea with hypoxia and/or findings on imaging) were observed in 6/45 (13%) pts at 180 mg QD. These symptoms needed urgent intervention. The respiratory symptoms were not observed at 90 mg QD (n = 8) or in the lead-in dose cohort (n = 19; initiated at 90 mg QD, escalated to 180 mg QD after 1 wk). Therefore, further enrollment with this dose escalation scheme, and an additional cohort of 90 mg QD without escalation were being added. Among 38 evaluable ALK+ NSCLC pts who had prior crizotinib, 24 (63%) reported response, including one CR. Six of 10 pts enrolled with untreated or progressing brain metastases showed response in brain, including 4 with complete resolution; 2 stable disease, 2 progressed; AP26113 has promising anti-tumor activity in pts with crizotinib-resistant ALK+ NSCLC, including pts with brain metastases. A randomized phase 2 trial of AP26113 comparing 90 mg QD vs. 90 mg QD escalating to 180 mg QD in crizotinib-resistant ALK+ NSCLC was planned.

#### ASP3026

ASP3026 is an oral, selective, potent, ATP competitive small molecule inhibitor of ALK with an IC50 of 3.5nM for ALK [95,96]. A phase I dose escalation trial was initiated to evaluate the safety and clinical activity of ASP3026 in patients with advanced malignancies (excluding leukemia) (NCT01284192). Thirty patients were enrolled in the dose escalation (25–800 mg) part of the study. The most common AEs were constipation, vomiting, nausea and abdominal pain. Grade 3 rash and ALT/AST elevation were dose-limiting. The MTD was established as 525 mg QD with a promising safety and pharmacokinetic (PK) profile in patients with advanced malignancies [95]. Patients (pts) with advanced solid tumors were treated with ASP3026 under fasting conditions without interruption in 3 + 3 dose escalation, “fast follower” phase I trial [97]. The cohorts received ASP3026 from 25 to 800 mg once daily (QD). At the last report for 2014 ASCO annual meeting, 33 patients were enrolled in the dose escalation phase, including 3 ALK+ pts, The phase Ib expansion cohort enrolled another 13 ALK+ pts [total pts N = 46; median (range) age = 61 (19–77) years]. Nausea /vomiting, rash and ALT/AST elevation were dose limiting toxicities. The MTD was 525 mg daily which became the recommended phase 2 dose (RP2D). The most common AEs were fatigue, and GI toxicities. Of 15 pts with ALK+ NSCLC who failed prior crizotinib, 7 (44%) had a PR and 8 had stable disease. The “fast follower” design allowed enrollment of ALK+ pts who achieved PR before the MTD of 525 mg QD was identified. Clinical activity was seen in the phase I trial in ALK+ NSCLC pts who failed crizotinib (NCT01401504).

#### ALK inhibitors in early phase development

X-376 and X-396 are novel, potent and specific ALK inhibitors with an aminopyridazine-based structure shared by crizotinib. X-396 had a 10 fold higher potency as compared to crizotinib across various cancer cell lines [98]. In addition, X-396 seems to be active against ALK mutants resistant to crizotinib. It has also been shown to penetrate blood–brain barrier.

TSR-011 is a dual ALK/TRK inhibitor developed by Tesaro, Inc., Waltham, MA, USA. It is currently recruiting in a phase I/IIa trial (NCT02048488) [99]; Preliminary results showed a dose range of 30–480 mg, with the DLTs to be dysaesthesia and QTc prolongation. PK modeling has identified 60 mg to have minimal peak exposure with sustained trough concentrations above IC50 required for ALK inhibition. Three of 5 patients with ALK+ NSCLC have achieved PR.

CEP-37440 is a dual ALK/FAK inhibitor currently under investigation in a phase I trial (NCT01922752). Focal adhesion kinase (FAK) is a ubiquitously expressed non-receptor tyrosine kinase implicated in cell adhesion and cell membrane-extracellular matrix interactions. It is thought to be involved in the carcinogenesis of colon cancer and other tumors of epithelial origin [100,101].

NMS-E628 (RXDX-101) is an orally available ALK/ROS-1 Inhibitor. It has been shown to induce complete regression of NSCLC and ALK+ leukemia cells in vitro and in vivo [102]. Currently a phase I/IIa trial studying RXDX-101 (NCT02097810) in locally advanced and metastatic solid tumors is in the recruitment phase.

PF-06463922 is a novel dual inhibitor of ALK/ROS1 with unusual activity against ROS1 kinase. PF-06463922 has IC50 values ranging from 0.1 nM to 1 nM toward ROS kinase inhibition. It was shown to be active across a panel of cell lines harboring ROS1 fusion variants including CD74-ROS1, SLC34A2-ROS1 and Fig-ROS1. This agent was developed to increase CNS availability and widen the spectrum of activity from crizotinib [103]. It has also been shown to overcome the crizotinib resistant CD74-ROS1^G2032R^ mutant. In addition, cyclin D1 was found to be suppressed by this inhibitor in vitro [104]. A phase I/IIa trial (NCT01970865) is currently in the recruitment phase.

## Conclusion and future directions

More and more novel agents for targeted therapy of lung cancers are rapidly migrating from bench to bedside [19,89,105-110]. Over the past decade, multiple small molecule inhibitors with activity against ALK and related oncoproteins have been developed [3]. Two of them, crizotinib and ceritinib, have gone on to get FDA approval for clinical use in locally advanced and metastatic NSCLC. More agents with improved safety, selectivity, potency and efficacy profiles are in the pipeline. Dual inhibitors targeting ALK as well as EGFRm, TRK, FAK, or ROS1 are under active clinical development. These agents may have the potential to concur the emerging mutants which become resistant to crizotinib and other agents in refractory and relapsed NSCLC and other solid tumors.
